# Prevalence of Epstein-Barr virus, human papillomavirus, cytomegalovirus and herpes simplex virus type 1 in patients with diabetes mellitus type 2 in south-eastern Poland

**DOI:** 10.1371/journal.pone.0222607

**Published:** 2019-09-24

**Authors:** Jakub Dworzański, Bartłomiej Drop, Ewa Kliszczewska, Małgorzata Strycharz-Dudziak, Małgorzata Polz-Dacewicz

**Affiliations:** 1 Masovian Specialist Hospital, Radom, Poland; 2 Department of Information Technology and Medical Statistics, Medical University of Lublin, Lublin, Poland; 3 Department of Virology, Medical University of Lublin, Lublin, Poland; 4 Chair and Department of Conservative Dentistry with Endodontics, Medical University of Lublin, Lublin, Poland; Arizona State University, UNITED STATES

## Abstract

A microbiota is a complex ecosystem of microorganisms consisting of bacteria, viruses, protozoa, and fungi living in different niches of the human body, which plays an essential role in many metabolic functions. Modifications in the microbiota composition can lead to several diseases, including metabolic disorders. The aim of this study was to analyze the prevalence of four viruses which can cause persistent infections–Epstein-Barr virus (EBV), human papillomavirus (HPV), cytomegalovirus (CMV), and herpes simplex virus type 1 (HSV-1) in patients with diabetes mellitus type 2 (DM2). Blood, saliva and oral swabs were collected from all the study participants. The nested-PCR technique was used to detect the viral DNA. DNA of at least one virus was detected in 71.1% of diabetic patients and in 30% of individuals without diabetes. In patients with diabetes EBV DNA was detected the most frequently (25.4%), followed by HPV– 19.1%, HSV– 10.4% and CMV– 5.2%. A higher percentage of EBV+HPV co-infection was found among men (30.8%). EBV DNA was statistically more often detected in patients living in rural areas (53.7%), while HPV (91.5%) and EBV+HPV co-infection (22.2%) prevailed among patients from urban areas. In patients with a DM2 history longer than 10 years viral infection was detected more frequently. The prevalence of EBV, HPV and the EBV+HPV co-infection was significantly higher in diabetic patients than in individuals without diabetes. The frequency of these infections depended on the duration of the disease (DM2).

## Introduction

A microbiota is a complex ecosystem of microorganisms consisting of bacteria, viruses, protozoa and fungi living in different niches of the human body, such as gastrointestinal tract, skin, oral cavity, respiratory tract and vagina. This ecosystem plays a key role in many metabolic functions, including glucose modulation. Modifications in the microbiota composition can lead to several diseases, including metabolic disorders [1].

The human microbiome consists of more than 100 trillion microbial cells [2]. Over 70% of microbiota is found in the gastrointestinal tract–in the stomach and in the small and large intestine, where the concentration of microorganisms is the highest [1,3]. Changes in the microbiome composition were observed in various diseases [4,5]. Studies concerning microbiome available in the literature concentrate mainly on bacteria and archaea [6,7]. However, the analysis of virome is a kind of “dark matter” in the microbial community [8].

It is estimated that viral sequences constitute approximately 42% of the human genome [4]. Handley's studies reported that the human virome consists of viruses that affect both eukaryotic and prokaryotic cells [4]. Viruses can integrate their genomes into host genomes, and also activate the immune system due to chronic infections [9]. Viral DNA and RNA stimulate the production of interferons and other cytokines, especially pro-inflammatory cytokines such as IL-10, which can modulate the immunological system of the host and contribute to the development of various diseases and secondary infections, with chronic infections as crucial in this process. Many viruses enter latency state, in which their genetic material is integrated with DNA of the infected cells. One of such groups of viruses playing important role in human pathophysiology is the Herpesviridae family (herpes simplex virus–HSV, Epstein-Barr virus–EBV, cytomegalovirus–CMV) [6,10]. A virome can also comprise human papilloma viruses (HPV), which can induce asymptomatic skin and mucosal infections [11].

Patients with diabetes mellitus type 2 (DM2) are more susceptible to infections as hyperglycaemia increases the virulence of different microorganisms [12]. Approximately 415 million people were diagnosed with DM2 in 2015 and there is expected increase to 642 million DM2 patients by the year 2040, therefore, this disease is considered to be the first non-infectious epidemic in the world and major public health concern of the 21st century [13,14]. Moreover, diabetes may cause many complications and can promote tumor progression and increase the risk of malignant carcinomas [15].

The aim of this study was to analyze the prevalence of four viruses widespread in the human population, which can cause persistent infections–EBV, HPV, CMV, and herpes simplex virus type 1 (HSV-1) in patients with DM2. The presence of these viruses in diabetic patients was analyzed regarding epidemiological data.

## Materials and methods

### Patients

The examination involved 173 adult patients treated for DM2 at the Department of Internal Diseases of the Masovian Specialist Hospital in Radom, Poland (south-eastern Poland). The control group comprised 50 individuals without diabetes (patients treated at the Department of Internal Diseases of the Masovian Specialist Hospital in Radom, Poland due other diseases than DM2).

The study was approved by the Medical University of Lublin Ethics Committee, and is in accordance with the GCP regulations (No. KE-0254/135/2017, 25 May 2017). Informed written consent was collected from all participants.

### Clinical specimens

Blood, saliva and oral brush swabs were collected from the patients and controls. HPV and HSV DNA was detected in the swab material, while EBV and CMV DNA in the saliva. The presence and titer of antibodies against HSV-1 (anti-HSV-1) were detected in the serum of all the subjects.

#### Saliva collection

About 5 ml of non-stimulated whole saliva was collected. The saliva samples were centrifuged at 1500 rpm at room temperature for 10 min, and then diluted (1:1) in PBS and frozen at -80°C until their analysis.

#### Serum collection

Venous blood samples from both the patients and the controls were centrifuged at 1500 rpm at room temperature for 15 min, followed by serum collection and frozen at -80°C until its analysis.

#### Oral brush swab collection

A cytobrush was rubbed with medium pressure over the premolar and molar region of the cheeks 5 times, turned and rubbed another 5 times. This procedure was performed both on the right and left cheeks. A cytobrush was then placed in Eppendorf tube and frozen at -80°C until the analysis.

### Molecular methods

DNA from the saliva and oral swabs was isolated with the use of the QIAamp kit (Qiagen, Germany).

#### DNA extraction from saliva and from brush swabs

DNA isolation was performed using the QIAamp DNA Mini Kit (Qiagen, Hilden, Germany) according to the manufacturer’s instructions. The efficiency and purity of the obtained eluate were checked using the Epoch (Biotek) spectrophotometer. The measurement was performed on a Take 3 plate (Biotek Instruments, Winooski, Vermont, US) using Microplate Reader Software Gen 5.2.0 (Biotek Instruments, Winooski, Vermont, US).

#### EBV DNA detection

EBV DNA detection and the amplification of the Epstein–Barr nuclear antigen 2 (EBNA-2) gene (the nested PCR) were performed as previously described [16].

The nested PCR was carried out for amplification of Epstein-Barr nuclear antigen 2 (EBNA-2). The sequence of primers used for PCR was as follows: outer pair 5’–TTT CAC CAA TAC ATG ACC C– 3’, 5’–TGG CAA AGT GCT GAG AGC AA– 3’ and inner pair 5’–CAA TAC ATG AAC CRG AGT CC– 3’, 5’–AAG TGC TGA GAG CAA GGC MC– 3’.

#### HPV detection and genotyping

HPV genotyping was performed as previously described [17].

HPV detection and genotyping was performed using the INNO-LiPA HPV Genotyping Extraassay (Innogenetics N. V, Gent, Belgium; no cat. 81063) according to the manufacturer’s protocol.

#### CMV and HSV detection

The concentrations of PCR reaction components, and also the amplification and detection of CMV DNA and HSV DNA was described previously [18].

CMV: a highly conservative region, i.e. the UL54 gene fragment, was amplified. The final product of PCR is 78bp. The nested-PCR technique was employed. The starters sequence: 1^st^ round of PCR: CMV-1F 5’–CGG GTC ATC TAC GGG GAC ACG GA– 3’, CMV-1R 5’–ACT TTG CCG ATG TAA CGT TTC TT– 3’; 2^nd^ round of PCR: CMV-2F 5’–GGG CCC AGC CTG GCG CAC TA– 3’, CMV-2R 5’–GAC GAA GAC CTT TTC AAA CTC– 3’.

HSV: a fragment of the gene encoding glycoprotein D (gD) was amplified. The final product of PCR is 280 bp. The nested-PCR technique was employed. The starters sequence:

1^st^ round of PCR: HSV-1F 5’–ATC CGA ACG CAG CCC CGC TG– 3’, HSV-1R 5’–TCC GGC GGC AGC AGG GTG CT– 3’; 2^nd^ round of PCR: HSV-2F 5’–GCG CCG TCA GCG AGG ATA AC– 3’, HSV-2R 5’–AGC TGT ATA CGG CGA CGG TG– 3’.

### Serological methods

In the collected serum samples, serological tests for anti-HSV-1 were conducted (ELISA aasay), using the commercially available NovaLisa Herpes Simplex Virus 1 (HSV1) IgG (Nova Tec Immunodiagnostica, Germany; Product No. HSV1G0500) kits. The level of HSV antibodies were expressed in NovaTec Units = NTU.

### Statistical analysis

Statistical analysis was performed using Pearson’s chi-square test, Fisher’s exact test for small groups, as well as the Mann-Whitney U test and the Kruskal Wallis test. The statistical significance was defined as p < 0.05.

## Results

Out of 173 patients with DM2 initially qualified to the study, 71.1% (123 patients) were found to have at least one virus, compared to 30.0% in the control group (p<0.001). Therefore, further analysis included 123 patients with at least one virus detected. The epidemiological characteristics of the patients with diabetes and individuals without diabetes is presented in Table 1.

**Table 1:** Epidemiological characteristics of patients with and without diabetes.

	Patients group N = 173		Control group N = 50		p
	N	%	N	%	
**Sex**					
Male	73	42.1	21	42.0	>0.05
Female	100	57.8	29	58.0	
**Age**					
20–39	24	13.8	7	14.0	>0.05
40–59	63	36.4	18	36.0	
60+	86	49.7	25	50.0	
**Place of residence**					
Urban	100	57.8	29	58.0	>0.05
Rural	73	42.2	21	42.0	
**Smoking**					
Yes	107	61.8	31	62.0	>0.05
No	66	38.2	19	38.0	
**Alcohol abuse**					
Yes	100	57.8	29	58.0	>0.05
No	73	42.2	21	42.0	
**BMI**					
18.5–24.9	29	16.8	-	-	-
25–29.9	43	24.9	-	-	
30–39.9	101	58.4	-	-	
**Duration of diabetes (years)**					-
1–5	38	22.0	-	-	-
6–10	44	25.4	-	-	
>10	91	52.6	-	-	

BMI—body mass index, N–number of patients

Pearson’s chi-square test

In terms of sex, age, place of residence, smoking, and alcohol consumption, the control group matched the study group and did not reveal statistically significant differences in comparison with the cases. Therefore, the socio-demographic factors had no effect on the values of the investigated variables.

**Fig 1:**
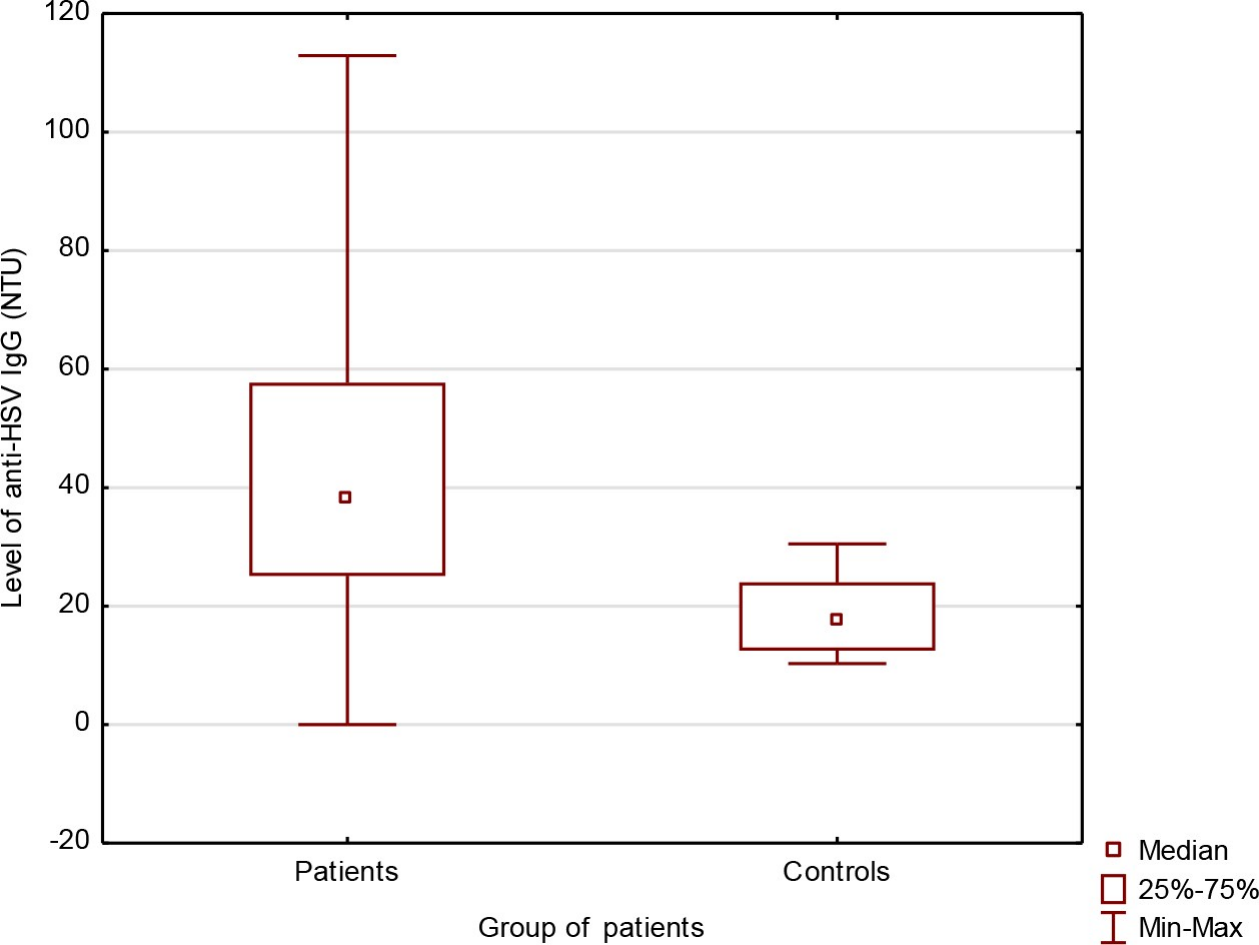
Prevalence of EBV, HPV, CMV, HSV and co-infection EBV+HPV in diabetic patients. * statistically significant p<0.05 (Pearson’s chi square-test).

In DM2 patients EBV DNA was detected the most frequently (25.4%), followed by HPV– 19.1%, HSV– 10.4% and CMV– 5.2% (Fig 1).

Infection prevalence was not correlated with age. However, EBV+HPV co-infection was significantly more frequent among men (30.8%) than in women (4.2%). EBV DNA was statistically more often detected in patients living in rural areas (53.7%), while HPV (91.5%) and co-infection (22.2%) prevailed among patients from urban areas (Table 2).

**Table 2:** Prevalence of EBV, HPV, CMV and HSV by sex, age, place of residence and duration of diabetes.

	EBV		HPV		CMV		HSV		EBV+HPV	
	N	%	N	%	N	%	N	%	N	%
**Sex**										
Male	17	32.7	10	19.2	5	9.6	4	7.7	16	30.8
Female	27	38.0	23	32.4	4	5.6	14	19.7	3	4.2
p	0.5419		0.1035		0.4022		0.0623		0.0004*	
**Age**										
20–39	5	11.4	3	9.1	1	11.1	4	22.2	4	21.1
40–59	17	38.6	12	36.4	2	22.2	4	22.2	10	52.6
60+	22	50.0	18	54.5	6	66.7	10	55.6	5	26.3
p	0.7159		0.4191		0.4341		0.1917		0.0512	
**Place of residence**										
Urban	22	26.8	16	91.5	6	7.3	12	14.6	16	22.2
Rural	22	53.7	17	41.6	3	7.3	6	14.6	3	5.9
p	0.0034*		0.0034*		0.9999		0.9999		0.0351*	
**Duration of diabetes (years)**										
1–5	13	29.5	3	9.1	0	0	5	27.8	1	5.3
6–10	9	20.0	9	27.3	2	22.2	6	33.3	2	10.5
>10	22	50.0	21	63.6	7	77.8	7	38.9	16	84.2
p	0.00004*		**10**^−6^*		0.0349*		0.4963		0.0376*	

*statistically significant (Pearson’s chi-square test)

EBV–Epstein-Barr Virus, HPV–Human Papillomavirus, CMV–Cytomegalovirus, HSV—Herpes Simplex Virus

Infection prevalence also depended on DM2 duration. In patients with a DM2 history longer than 10 years viral infection was detected more frequently. In EBV(+) patients 50% comprised patients with diabetes lasting longer than 10 years, in HPV (+)– 63.6%, in CMV(+)– 77.8% and EBV+HPV co-infection– 84.2%.

The analysis revealed that in diabetic patients with higher BMI, DNA of all examined viruses and co-infection was more frequently detected compared with patients with normal BMI (Table 3).

**Table 3:** Prevalence of EBV, HPV, CMV and HSV by BMI index.

BMI	EBV		HPV		CMV		HSV		EBV+HPV	
	N	%	N	%	N	%	N	%	N	%
Normal	7	15.9	4	12.1	0	0	1	5.5	1	5.3
Overweight	15	34.1	5	15.2	1	11.1	3	16.7	3	15.8
Obesity	22	50.0	24	72.7	8	88.9	14	77.8	15	78.9
p	0.5061		0.0360*		0.0344*		0.0385*		0.0374*	

*statistically significant (Mann-Whitney U test)

EBV–Epstein-Barr Virus, HPV–Human Papillomavirus, CMV–Cytomegalovirus, HSV—Herpes Simplex Virus, N–number of patients, BMI-body mass index

**Fig 2:**
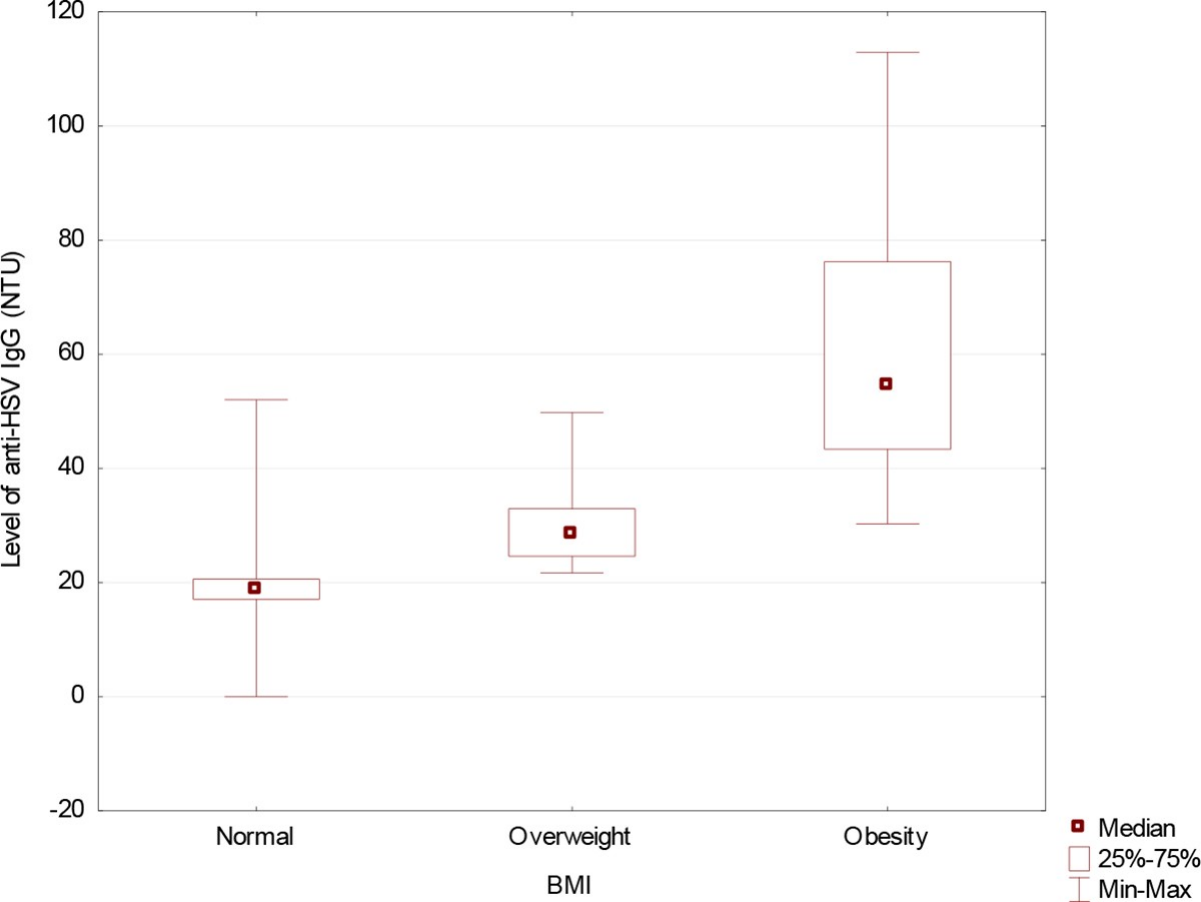
Level of anti-HSV IgG in diabetic patients compared to the controls. (Mann-Whitney U-Test: Z = 7.985016; p = 10^−4^) HSV IgG—IgG antibodies herpes simplex virus, NTU—NovaTec Units.

**Fig 3:**
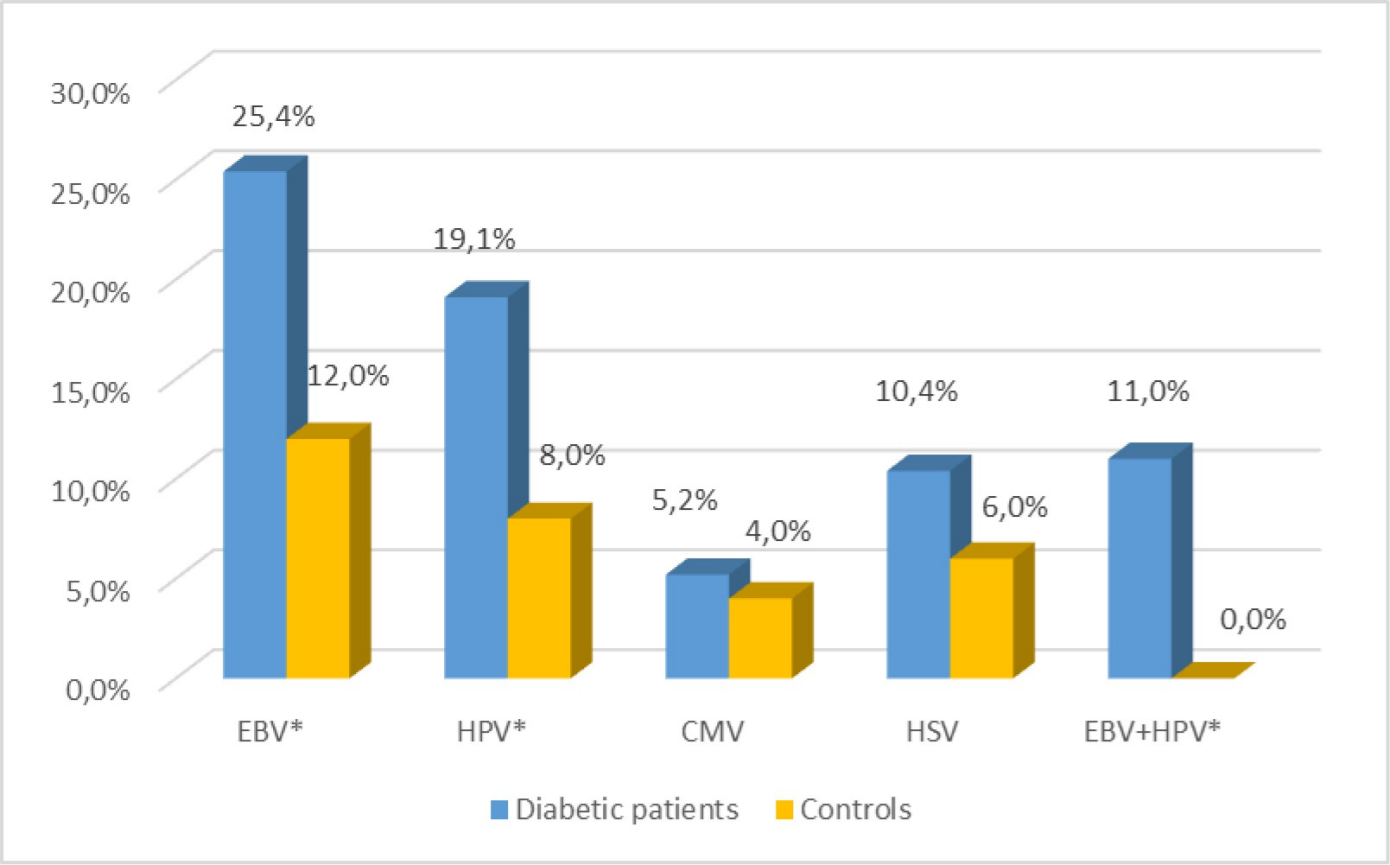
Anti-HSV-1 antibody level by BMI index among diabetic patients. (Kruskal-Wallis Test; H = 82.57; p = 10^−4^) HSV IgG—IgG antibodies herpes simplex virus, BMI-body mass index, NTU—NovaTec Units.

The level of anti-HSV IgG antibodies were significantly higher in the serum of diabetic patients than in controls (Fig 2). Obese patients was the group with the highest infection percentage (Fig 3).

## Discussion

Persistent viral infections, involving long interaction time between the virus and the infected cell, are particularly significant from the clinical point of view. They are latent infections with potential periodic reactivation [19,20]. A typical example is HSV-1: following a primary acute or subclinical infection, this virus moves through the sensory nerves and attains latency by settling in the trigeminal root [21]. A range of factors can cause reactivation of infection and manifest with typical herpes symptoms. The fact that two-thirds of the world's population is infected with HSV-1 proves the excellent survival strategy of this virus [22].

Persistent infections involving various viruses affect the overall immunity of the patient. In our study EBV DNA was the most prevalent in the group of diabetic patients (35.9%). EBV is widespread in human population and is easily transmitted through direct contact, so people with long-term glycaemia are more likely to become infected than healthy population.

There are few reports in the literature about the relationship between DM2 and viral infections, as most studies concern type 1 diabetes (DM1). Some researches revealed that changes in the intestinal microbiome are associated with the development of DM1 [20,23]. It is suggested that enterovirus, cytomegalovirus, mumps virus, parvovirus, rubella virus, and rotaviruses may be crucial in that process. Lontchi-Yimagou et al. [24] showed that HHV-8 infections are associated with low insulin secretion. Moreover, differences in the ORF 26 sequence were found in 30% of the HHV-8 strains isolated from lymphocytes, prompting the authors to hypothesis the existence of a new HHV-8 virus sub-type [1,3]. It was also proved that DM2 is more likely to occur in patients with chronic hepatitis C virus [25].

Therefore, our studies on the prevalence of selected viruses in the Polish population can be considered pioneering. They revealed that DNA of at least one virus was statistically more frequently isolated in diabetic patients (70.3% compared to 30% in the control group), with EBV DNA and HPV DNA being the most prevalent. The prevalence of infections also depended on duration of the disease. The level of anti-HSV IgG antibodies was significantly higher among patients with DM2 as compared to the controls. Some researchers suggest that anti-HSV IgG antibodies level is associated with the incidence of diabetic atherosclerosis, and that CMV can be a risk factor in the development of this disease [26].

Both diabetes and cancer are diseases of modern civilization and their incidence has been increasing globally. Some tumors develop more frequently in diabetic patients, including especially those with DM2 [27]. Potential risk factors are similar for diabetes and cancer. Age, sex, race and ethnicity are non-modifiable risk factors, independent of the person's life style. The factors controlled by humans include physical activity, diet, alcohol consumption, and smoking. A number of studies revealed a correlation between obesity and the development of DM2, cardiovascular diseases, and liver cancer [28].

Overweight, one of the most widespread lifestyle-related health problems, was rated third among the most serious disease risk factors in 2013, both for the Polish population, and for the populations of Central and Western European countries [29]. According to WHO data, over 60% of men and over 50% of women in the European Union were either overweight or obese [30]. Epidemiological studies conducted in the United States revealed a correlation between body weight and the level of anti-HSV-1 antibodies [31]. In our studies, the level of anti-HSV-1 antibodies among diabetic patients was also significantly higher than in the controls. Larsen et al. [32] demonstrated that DM2 is associated with changes in the intestinal microbiota composition, indicating that both overweight and diabetes are associated with different groups of that microbiota. Interesting results were obtained in the analysis of BMI as a factor in the prevalence of HSV-1 DNA. HSV-1 DNA was found in 77.8% of obese subjects, and the level of anti-HSV-1 IgG antibodies was higher among obese and overweight patients compared to those with normal BMI values.

Our studies revealed that 5.7% of diabetic patients had also cancer disease. However, these were isolated cases of multi-organ tumors, which made it impossible to perform an epidemiological analysis regarding the potential relationship between the incidence of diabetes and cancer. This issue might be the subject of future studies involving larger populations. Oncogenesis is a complex and multi-stage process in which a normal cell changes its phenotype into a neoplastic one. Diabetes can contribute to neoplastic transformations through a range of mechanisms, with hyperinsulinaemia, hyperglycaemia, insulin resistance, or chronic inflammation being the potential underlying factors [33]. The immune dysfunction co-occurring with diabetes can cause the reactivation of some latent infections associated with high incidence and mortality rates. Insulin and glucose concentrations support the migrations of neoplastic cells and contribute to the in-vitro growth of neoplastic cell lines [34]. According to the literature data, diabetes involves an increased risk of liver, pancreatic, endometrium, large-intestine and mammary-gland tumors, while a reverse correlation applies to the incidence of prostate cancer [35]. The viruses investigated in this study belong to a group of human oncogenic viruses, which may lead to cancer development. International Agency for Cancer Research included both EBV and HPV in the viruses causing neoplastic diseases [36,37]. It is estimated that viral infections contribute to 15–20% of all human cancers such as prostate cancer, breast cancer and brain cancer [38].

Among the diabetic patients included in the study, EBV was the most often detected (35.9%). There is a proved connection between this virus and the development of various cancers, e.g. nasopharynx cancer and stomach cancer [39,40]. Papilloma viruses represent another significant group–HPV DNA was detected in 26.8% of diabetic patients. HPV 16 genotype was found only in three patients and mixed HPV types with low oncogenic risks were most common among the remaining patients. HPV is a proved etiologic factor in the development of cervical cancer (HPV18), as well as oral and pharyngeal cancer (mainly HPV16) [41–44]. Thus, the results suggest that diabetic patients might be at a higher risk of developing various malignancies due to high rates of oncogenic viral infections.

Our study had some limitations as the subject group was too small to allow an in-depth epidemiological analysis, so future studies should involve larger groups of patients. However, the results provide a general direction for research to establish how long-term diabetes influences the incidence of neoplastic diseases, to assess the relationship between latent viral infections, overweight and obesity, and the development of neoplastic diseases as well as to determine the impact of the investigated viral infections on the clinical picture of DM2 and especially on its control.

## Conclusions

The prevalence of EBV, HPV and the EBV+HPV co-infection was significantly higher in diabetic patients than in the individuals without diabetes. The frequency of these infections depended on the duration of the disease. HSV DNA was detected in 77.8% of the obese patients. The level of anti-HSV antibodies was the highest among the patients with high BMI. In DM2 patients with obesity, HPV, CMV, HSV infection and EBV+HPV co-infection was detected significantly more frequently.

## References

[1] Pascale A, Marchesi N, Marelli C, Coppola A, Luzi L, Govoni S, et al Microbiota and metabolic diseases. Endocrine. 2018; 61:357–371. 10.1007/s12020-018-1605-5 29721802

[2] Glasner ME. Finding enzymes in the gut metagenome. Science. 2017; 355:577–578. 10.1126/science.aam7446 28183934

[3] Parker MT. An Ecological Framework of the Human Virome Provides Classification of Current Knowledge and Identifies Areas of Forthcoming Discovery. Yale J Biol Med. 2016; 89:339–351. 27698618 PMC5045143

[4] Handley SA. The virome: a missing component of biological interaction networks in health and disease. Genome Med. 2016; 8:5 10.1186/s13073-015-0258-8 27037032 PMC4818473

[5] Houghton D, Hardy T, Stewart C, Errington L, Day CP, Trenell MI, et al Systematic review assessing the effectiveness of dietary intervention on gut microbiota in adults with type 2 diabetes. Diabetologia. 2018; 61:1700–1711. 10.1007/s00125-018-4632-0 29754286 PMC6061157

[6] Nikolich-Zugich J, Goodrum F, Knox K, Smithey MJ. Known unknowns: how might the persistent herpesvirome shape immunity and aging?. Curr Opin Immunol. 2017; 48:23–30. 10.1016/j.coi.2017.07.011 28780492 PMC5682194

[7] Carding SR, Davis N, Hoyles L. Review article: the human intestinal virome in health and disease. Aliment Pharmacol Ther 2017; 46:800–815. 10.1111/apt.14280 28869283 PMC5656937

[8] Han M, Yang P, Zhong C, Ning K. The Human Gut Virome in Hypertension. Front Microbiol. 2018; 9:3150 10.3389/fmicb.2018.03150 30619215 PMC6305721

[9] Virgin HW. The virome in mammalian physiology and disease. Cell. 2014; 157:142–150. 10.1016/j.cell.2014.02.032 24679532 PMC3977141

[10] Boppana SB, Fowler KB. Persistence in the population: epidemiology and transmission In: Arvin A., Campeadelli-Fiuwe G., editors. Human Herpesviruses: Biology, Therapy and Immunoprophylaxis. Cambridge: Cambridge University Press; 2007 p. 36. 21348071

[11] Ogilvie LA, Jones BV. The human gut virome: a multifaceted majority. Front Microbiol. 2015; 6:918 10.3389/fmicb.2015.00918 26441861 PMC4566309

[12] Casquiero J, Casquiero J, Alves C. Infections in patients with diabete mellitus; A review of pathogenesis. Indian. J Endocrinol Metab. 2012; S27–36. 10.4103/2230-8210.94253 22701840 PMC3354930

[13] Ogurtsova K, da Rocha Fernandes JD, Huang Y, Linnenkamp U, Guariguata L, Cho NH, et al IDF Diabetes Atlas: Global estimates for the prevalence of diabetes for 2015 and 2040. Diabetes. Res Clin Pract. 2017; 128:40–50. 10.1016/j.diabres.2017.03.024 28437734

[14] Guariguata L, Whiting DR, Hambleton I, Beagley J, Linnenkamp U, Shaw JE. Global estimates of diabetes prevalence for 2013 and projections for 2035. Diabetes Res Clin Pract. 2014; 103:137–49. 10.1016/j.diabres.2013.11.002 24630390

[15] Oikonomou E, Mourouzis K, Fountoulakis P, Papamikroulis GA, Siasos G, Antonopoulos A, et al Interrelationship between diabetes mellitus and heart failure: The role of peroxisome proliferator-activated receptors in left ventricle performance. Heart Fail Rev. 2018; 23:389–408. 10.1007/s10741-018-9682-3 29453696

[16] Polz-Dacewicz M, Strycharz-Dudziak M, Dworzanski J, Stec A, Kocot J. Salivary and serum IL-10, TNF-α, TGF-β, VEGF levels in oropharyngeal squamous cell carcinoma and correlation with HPV and EBV infection. Infect Agent Cancer. 2016; 11:45 10.1186/s13027-016-0093-6 27547238 PMC4992298

[17] Drop B, Strycharz-Dudziak M, Kliszczewska E, Polz-Dacewicz M. Coinfection with Epstein–Barr Virus (EBV), Human Papilloma Virus (HPV) and Polyoma BK Virus (BKPyV) in Laryngeal, Oropharyngeal and Oral Cavity Cancer. Int J Mol Sci. 2017; 18:E2752 10.3390/ijms18122752 29257122 PMC5751351

[18] Polz-Gruszka D, Stec A, Dworzański J, Polz-Dacewicz M. EBV, HSV, CMV and HPV an laryngeal and oropharyngeal carcinoma in Polish patients. Anticancer Res. 2015; 35:1657–61. 25750324

[19] Cadwell K. The virome in host health and disease. Immunity. 2015; 42:805–813. 10.1016/j.immuni.2015.05.003 25992857 PMC4578625

[20] Virgin HW, Wherry EJ, Ahmed R. Redefining chronic viral infection. Cell. 2009; 138:30–50. 10.1016/j.cell.2009.06.036 19596234

[21] Pellett PE, Roizman B. Herpesviridae In: Knipe DM, Howley P. editors. Fields Virology. Philadelphia:. Wolters Kluwer Health/Lippincott Williams & Wilkins; 2013 p. 1802–22.

[22] Looker KJ, Margaret AS, May MT, Turner KME, Vickerman P, Gottlieb SL, et al Global and Regional Estimates of prevalent and incident Herpes Simplex Virus type 1 infections in 2012. PLoS One. 2015; 10:e0140765 10.1371/journal.pone.0140765 26510007 PMC4624804

[23] Needell JC, Zipris D. The role of the intestinal microbiome in type 1 diabetes pathogenesis. Curr Diab Rep. 2016; 16:89 10.1007/s11892-016-0781-z 27523648

[24] Lontchi-Yimagou E, Legoff J, Nguewa JL, Boudou P, Balti EV, Noubiap JJ, et al Human herpesvirus 8 infection DNA positivity is associated with low insulin secretion: A case-control study in a sub-Saharan African population with diabetes. J Diabetes. 2018; 10:866–873. 10.1111/1753-0407.12777 29707905

[25] Zuwala-Jagiello J, Pazgan-Simon M, Murawska-Cialowicz E, Simon K. Influence of diabetes on circulating apoptotic microparticles in patients with chronic hepatitis C. In Vivo. 2017; 31:1027–1034. 10.21873/invivo.11165 28882977 PMC5656847

[26] Zhang J, Liu YY, Sun HL, Li S, Xiong HR, Yang ZQ, et al High Human Cytomegalovirus IgG Level is Associated with Increased Incidence of Diabetic Atherosclerosis in Type 2 Diabetes Mellitus Patients. Med Sci Monit. 2015; 21:4102–10. 10.12659/MSM.896071 26717490 PMC4699628

[27] Giovanuci E, Harlen DM, Archer MC, Bergenstal RM, Gapstur SM, Habel L, et al Diabetes and cancer. A consensus report. Diabetes Care. 2010; 33:1674–1685. 10.2337/dc10-0666 20587728 PMC2890380

[28] Yu J. Introduction. In Obesity, Fatty Liver and Liver Cancer In: Wong CC, Yu J, editors. Advances in Experimental Medicine and Biology, 1061. Singapore, Springer Nature; 2018 p. 1–2.

[29] Youn JC, Kim JY, Jung MK, Yu HT, Park SH, Kim IC, et al Analysis of cytomegalovirus-specific T-cell responses in patients with hypertension: comparison of assay methods and antigens. Clin Hypertens. 2018; 24:5 10.1186/s40885-018-0090-8 29568571 PMC5861653

[30] World Health Organization (WHO). Global Report on Diabetes. Geneva: WHO; 2016 10.2337/db15-0956

[31] Karjala Z, Neal D, Rohrer J. Association between HSV-1 seropositivity and obesity: data from the National Health and Nutritional Examination Survey, 2007–2008. PLoS One. 2011; 6:e19092 10.1371/journal.pone.0019092 21589933 PMC3092767

[32] Larsen N, Vogensen FK, van den Berg FWJ, Nielsen DS, Andreasen AS, Pedersen BK, et al Gut Microbiota in Human Adults with Type 2 Diabetes Differs from Non-Diabetic Adults. PLoS One. 2010; 5:e9085 10.1371/journal.pone.0009085 20140211 PMC2816710

[33] de Jong RGPJ, Peeters PJHL, Burden AM, de Bruin ML, Haak HR, Masclee AAM, et al Gastrointestinal cancer incidence in type 2 diabetes mellitus; results from a large population-based cohort study in the UK. Cancer Epidemiol. 2018; 54:104–111. 10.1016/j.canep.2018.04.008 29705628

[34] Masur K, Vetter C, Hinz A, Tomas N, Henrich H, Niggemann B, et al Diabetogenic glucose and insulin concentrations modulate transcriptome and protein levels involved in tumour cell migration, adhesion and proliferation. Br J Cancer. 2011; 104:345–52. 10.1038/sj.bjc.6606050 21179032 PMC3031898

[35] Garg SK, Maurer H, Reed K, Selagamsetty R. Diabetes and cancer: two diseases with obesity as a common risk factor. Diabetes Obes Metab. 2014; 16:97–110. 10.1111/dom.12124 23668396 PMC3904746

[36] IARC. Monographs on the evaluation of carcinogenic risks to humans A review of human carcinogens. Biological agents. Lyon: World Health Organization; 2012 p.255. PMC4781184 23189750

[37] Perera RA, Samaranayake LP, Tsang CSP. Shedding dynamics of Epstein-Barr virus: A type 1 carcinogen. Arch Oral Biol. 2010; 55:639–47. 10.1016/j.archoralbio.2010.06.009 20627195

[38] Alibek K, Kakpenova A, Baiken Y. Role of infectious agents in the carcinogenesis of brain and head and neck cancers. Infect Agent Cancer. 2013; 8:7–16. 10.1186/1750-9378-8-7 23374258 PMC3573938

[39] Tsao SW, Tsang CM, Lo KW. Epstein-Barr virus infection and nasopharyngeal carcinoma. Philos Trans R Soc Lond B Biol Sci. 2017; 372:20160270 10.1098/rstb.2016.0270 28893937 PMC5597737

[40] Bass AJ, Thorsson V, Shmulevich I, Reynolds SM, Miller M, Bernard B, et al Comprehensive molecular characterization of gastric adenocarcinoma. Cancer Genome Atlas Research Network. Nature. 2014; 513:202–9. 10.1038/nature13480 25079317 PMC4170219

[41] Garbuglia AR. Human papillomavirus in head and neck cancer. Cancers (Basel). 2014; 6:1705–1726. 10.3390/cancers6031705 25256828 PMC4190563

[42] Gillison ML, Castellsagué X, Chaturvedi A, Goodman MT, Snijders P, Tommasino M, et al Eurogin Roadmap: comparative epidemiology of HPV infection and associated cancers of the head and neck and cervix. Int J Cancer. 2014; 134:497–507. 10.1002/ijc.28201 23568556

[43] Jalouli J, Jalouli MM, Sapkota D, Ibrahim SO, Larsson PA, Sand L. Human papilloma virus, herpes simplex and Epstein-Barr virus in oral squamous cell carcinoma from eight different countries. Anticancer Res. 2012; 32:571–580. 22287747

[44] Syrjänen KJ, Syrjänen SM, Lamberg MA, Pyrhönen S. Human papillomavirus (HPV) involvement in squamous cell lesions of the oral cavity. Proc Finn Dent Soc. 1983; 79:1–8. 6306646

